# Workflow evaluation of a commercial deep learning-based image analysis tool for the quantification of angiogenesis *in vivo*

**DOI:** 10.1038/s41598-026-68133-1

**Published:** 2026-08-28

**Authors:** Christoph Raphael Buhr, Alexander Philippe Maas, Nadine Wiesmann-Imilowski, Juergen Brieger, Christoph Matthias, Benjamin Philipp Ernst, Anita Kloss-Brandstaetter, Philipp Herrmann, Jonas Eckrich

**Affiliations:** 1 Department of Otorhinolaryngology, University Medical Center Mainz, 55131 Mainz, 55131 Mainz, Germany; 2 Department of Otorhinolaryngology, University Medical Center Bonn (UKB), 53127 Bonn, Germany; 3 Department of Oral and Maxillofacial Surgery—Plastic Surgery, University Medical Center Mainz, 55131 Mainz, Germany; 4 Department of Otorhinolaryngology, University Medical Center Frankfurt, 60596 Frankfurt, Germany; 5 Department of Engineering & IT, Carinthia University of Applied Sciences, Villach, 9524 Austria; 6 Department of Ophthalmology, University Medical Center Bonn (UKB), 53127 Bonn, Germany

**Keywords:** Biological techniques, Computational biology and bioinformatics, Engineering, Medical research

## Abstract

Angiogenesis plays a crucial role in tumor development as well as in wound healing and integration of biomaterials. However, quantification of complex vascular networks across different in vivo models is time-consuming, labor-intensive, and prone to human error. Deep learning-based analysis (DLBA) appears to be a more efficient and reproducible method for vascular quantification . Yet, studies directly comparing its performance with human assessment remain limited. IKOSA CAM is a commercially available tool built for analysis of vascular networks. Here we evaluated IKOSA CAM against manual vessel tracing across three different angiogenesis models. Images obtained from three different in vivo angiogenesis models, the chorioallantoic membrane (CAM) assay, the rodent retina, and the dorsal skinfold chamber (DSC), were analyzed. The vascular network was analyzed using both manual tracing and the commercial deep learning-based image analysis tool IKOSA CAM. In addition, tracing results were reevaluated by human raters and the total workflow duration was assessed. Deep learning-based image analysis proved to be a time-efficient method for tracing and quantifying vascular networks. The level of agreement with manual human tracing varied among image types. The highest agreement between the two methods was observed for the CAM assay. The greatest differences occurred in the tracing of DSC branching points, as indicated by a downward trend in the Bland-Altman plots. Repeated analyses with the DLBA produced identical results for all tested angiogenesis models, confirming excellent reproducibility. Standardized vascular quantification remains a major challenge in experimental angiogenesis research. In this workflow validation study, the evaluated DLBA provided reproducible and time-efficient vessel quantification, particularly for the CAM assay, while showing limitations in the analysis of retinal and DSC images. Overall, DLBA represents a promising approach for standardized vascular analysis and may facilitate reproducible, high-throughput image quantification. Nevertheless, further validation using larger and more heterogeneous datasets is warranted.

**Supplementary Information:**

The online version contains supplementary material available at https://doi.org/10.1038/s41598-026-68133-1.

## Introduction

Angiogenesis is the process by which characterizes the formation of new blood vessels from a pre-existing vascular network. Growth and development of the vessel system are achieved by continuous sprouting and splitting from parent vessels orchestrated and influenced by a variety of organic and inorganic stimuli^1^. Thus, angiogenesis plays an elementary role for physiological processes such as growth and development of an organism, wound healing and ingrowth of biomaterials as well as pathological processes including tumor growth and metastasis^2^.

Accordingly, scientific interest in angiogenic processes is high and a large variety of models for the visual analysis of angiogenesis are available^3^. In vivo models take advantage of physiologically accessible vessels such as the cornea^4^, the retina^5^, or the vascular network on the outer ear or the tail of mice^6,7^, transparent zebrafish embryos^8^, or *Xenopus* tadpoles^9^. Alternatively, surgical exposure of the vascular system allows an unrestricted and repetitive insight into intensively vascularized tissues. Removal of the eggshell in the chorioallantois-membrane (CAM) assay as well as different chamber models enable repetitive analysis of the vascular network with in vivo microscopy. The CAM assay is an established model for in vivo angiogenesis research utilizing a fertilized egg. Removal of the overlaying eggshell allows insight onto its highly vascularized CAM^10,11^. Chamber models on the other hand use a surgically implanted glass window to allow visualization of vascular plexus of the specific tissues^12–17^.

Typical parameters of vascular analysis include the total vessel length (TVL), the functional vessel density (FVD), the vascular diameters (VD), as well as the amount of vessel branches and junctions of the vascular network^18^. Intravascular application of high molecular fluorescent dyes like Fluorescein Isocyanate (FITC) conjugated Dextran or Tetramethylrhodaminisothiocyanate (TRITC)-Dextran allows enhancement of the contrast between the vascular network and the surrounding tissue and is used to visualize vascular perfusion in fluorescence microscopy^13,19^.

For the evaluation of vascular networks manual counting as well as manual retracing of the vessels in a pre-defined region of interest (ROI) is routinely carried out. However, these methods are very time consuming and prone to human error. Semiautomated vascular analysis with image analysis software such as ImageJ^20^ or alternative software like AngioTool^21^ or the Metamorph Software with an angiogenesis module^22,23^ allow an accelerated analysis but often rely on a very intense contrast between the vascular network and the background. Thus they are prone to inconsistencies depending on the specific image quality and the thresholds applied for image analysis^24^. Apart from limitations for vascular analysis caused by image quality, time consumption may be the most relevant limitation. Manual labeling has obvious advantages like the ability to interpret and estimate the course of vessels in difficult conditions. Yet, especially when larger ROI or entire vascular networks are evaluated, the time requirement becomes a major limitation.

Deep learning-based image analysis (DLBA) employs artificial neural networks, most commonly convolutional neural networks (CNNs). Thus, it can automatically learn hierarchical image features directly from image data instead of relying on manually engineered descriptors^25,26^. In CNNs, convolutional layers progressively extract increasingly complex image characteristics, ranging from edges and textures to higher-level structural patterns, enabling robust object tracing, classification, and semantic segmentation. Compared to classical image-processing approaches, such as threshold-based segmentation, edge tracing, or handcrafted feature extraction, CNN-based methods are generally more robust to variations in image quality, contrast, illumination, and biological variability because relevant image features are learned directly from annotated training datasets rather than being predefined by the user^25,26^. In recent years, CNN-based approaches have increasingly been applied to the analysis of vascular networks. Such methods have been used for the segmentation of coronary angiograms or for the quantification of angiogenesis in established in vivo models, including tumour-induced angiogenesis and the CAM assay. These studies indicate that CNN-based segmentation can provide reproducible and observer-independent quantification of complex vascular structures, motivating a direct comparison with manual tracing as performed in the present study^27–29^.

DLBA approaches can broadly be categorized into supervised, semi-supervised, and self-supervised learning strategies. Supervised methods rely on manually annotated training datasets and currently represent the predominant approach for biomedical image segmentation, whereas semi-supervised approaches aim to reduce annotation effort by additionally exploiting unlabeled data^30^. The IKOSA CAM application (KOLAIDO GmbH, Altenrhein, Switzerland) belongs to the category of supervised CNN-based vessel segmentation methods. Its built-in feature “CAM Assay” facilitates such an AI-based analysis specifically trained for the tracing of vascular networks in CAM images. According to the manufacturer, the image analysis model is based on a CNN, utilizing an InceptionResNetv2 ^31^ as an encoder and a U-Net^32^-based structure as a decoder path. It was trained on both retinal fundus images and images of the CAM^27,31,32^.

The aim of this pilot validation study was to evaluate the workflow and the agreement between manual vessel annotation and the commercially available DLBA platform IKOSA CAM across three established angiogenesis models. Rather than benchmarking segmentation algorithms or establishing absolute accuracy, this workflow focused pilot study sought to assess the feasibility, reproducibility, and agreement of AI-assisted vessel quantification under representative experimental conditions usung manual annotation as the reference method.

## Materials and methods

### Study design and legal aspects

The specific experimental procedures were performed according to institutional and governmental guidelines and animal experiments were approved by the Landesuntersuchungsamt Rheinland-Pfalz (G 15-1-066) as well as (UCL in London; PPL 70/1279). All selected image files were obtained from preliminary experiments and from experiments already published by our work group^18^. Vessel tracing was performed with unedited images to ensure a consistent analysis pattern. All people involved in the experimental course and retrieval of the images were qualified and certified to perform experimental procedures on laboratory animals.

**Fig. 1:**
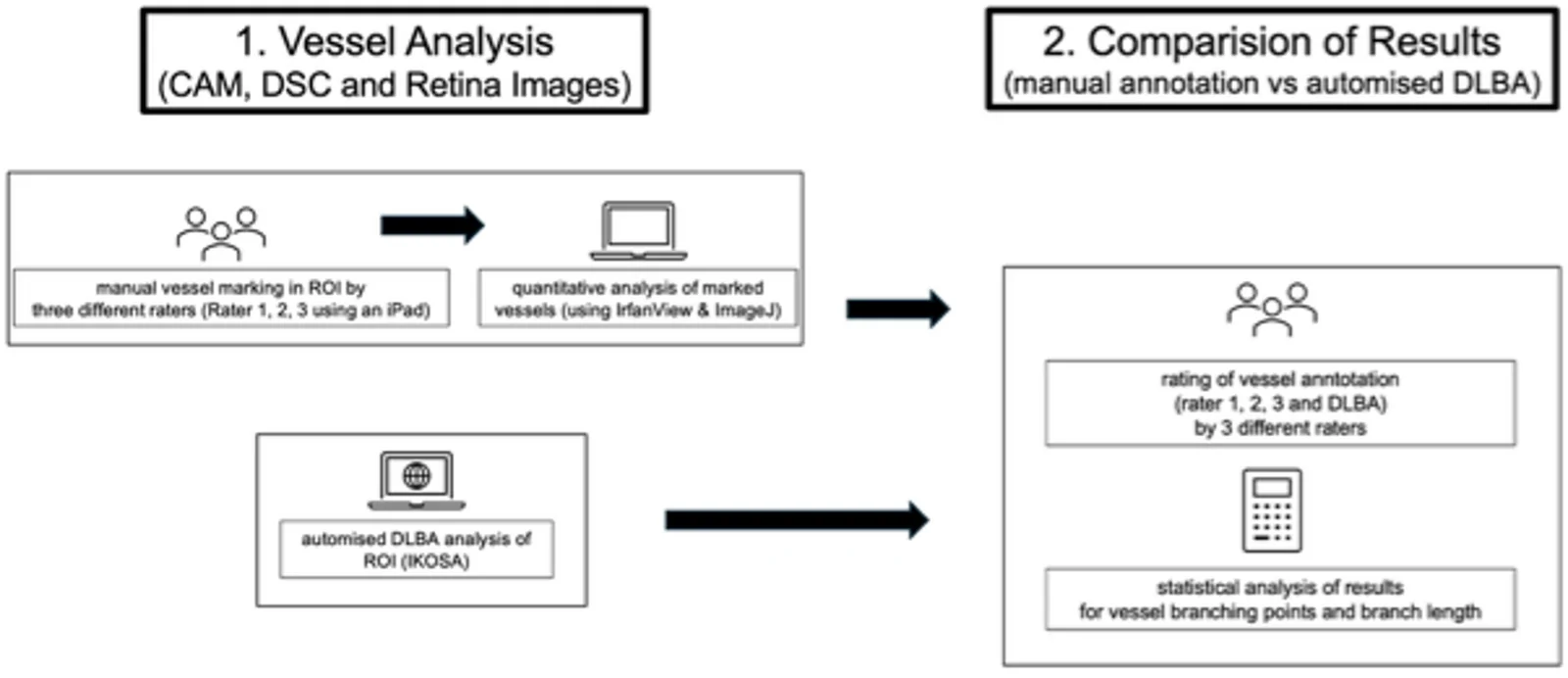
Workflow of the study.

**Fig. 2:**
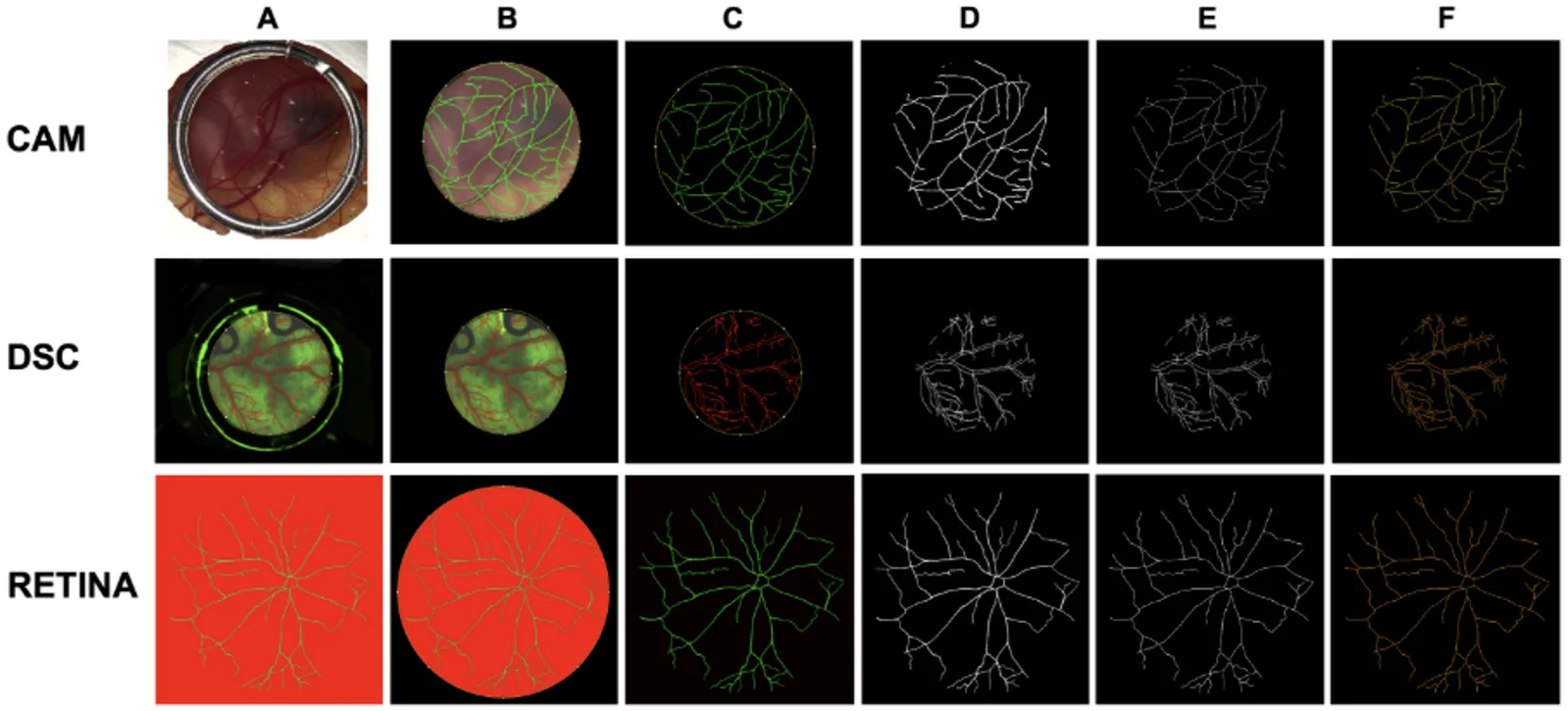
Step-by-step approach of manual tracing processed in ImageJ for each model. (**A**) Image with vessels marked by human raters. (**B**) Cropped region of interest (ROI). (**C**) Modified “color balance”, isolated visualization of the vessels and isolated “color channel” (green: CAM, RETINA; red DSC). (**D**) Binarized image. (**E**) Skeletonized image. (F) Output of Vascular analysis by ImageJ.

Vascular analysis was performed in three different angiogenesis models. More specifically, ten images of the CAM assay (chicken egg (Gallus gallus)), nine images of the mouse (*Mus musculus*) retinae (C57BL/6J) and ten images obtained with the dorsal skinfold chamber of C57BL/6J mice were analyzed. Images were processed as high resolution JPG files. For the CAM, all images were taken with the microscope’s built-in camera on day ten of incubation. DSC images s were captured on day 2 after chamber implantation. While CAM images were aquired as a single photograph (1920 × 1080 px) taken in 12.5-fold magnification, DSC images were automatically stitched together utilizing the plugin “Multi Image Array”. of our microscope's built in software Cell Sens Dimension (Olympus Deutschland GmbH, Hamburg, Germany). All images of the vascular network showed a “native” vascular network without any pro- or anti-vascular treatment. The workflow of the study is shown in Fig. 1:

The area of evaluation was declared as ROI (Fig. 2). Subsequently, all ROI were analyzed by IKOSA as well as by the different raters respectively as follows:

### Manual analysis

1. Manual vessel analysis: Three different raters traced the vessels in the defined ROI using an iPad 2020 8th Generation (Apple, California, USA) and an Apple Pencil 1st Generation (Apple, California, USA) utilizing the built-in “Edit Image” and “Markup” function. Depending on the image, the color with the greatest possible contrast to the image was selected. Specifically, the CAM images and the retina images were traced with green, while the dorsal skin chamber images (taken in the green fluorescent channel) were marked with red (Fig. 2A). All major vessels were marked. Only arteries, arterioles, veins, and venules were included in the manual annotation. Capillaries were excluded from the analysis. Capillaries were operationally defined as the smallest microvessels that could not be reliably traced over a continuous course and/or vessels exhibiting the characteristic *rouleaux* appearance in arrangement of erythrocytes within the vascular lumen and/or thin linear microvascular structures without a clear transition into larger vessels. Specifically, in the DSC images capillaries frequently show a linear course between skeletal muscle fibers. All people involved in the annotation process were trained in the same laboratory thus adhering to the same annotation principles. Upon annotation, the image subsequently saved as a jpg file. For the retinal images, due to the high proportion of white pixels in the dichromate image, further preparation before analysis was mandatory. Hence, images were adjusted after vessel tracing using the freeware program IrfanView (Version 4.60) (Fig. S2A). In IrfanView the option “Image”, “Decrease color depth” was selected. The number of colors was subsequently reduced to six, opting for “Use Floyd-Steinberg dithering” (Fig. S2B). After reduction of colors within the image “Image”, “Replace color” was selected and all colors except for the green markup were replaced with the color red (Fig. S2C).
2. All images were subsequently edited and analyzed using ImageJ^20^. First all areas outside the ROI were excluded by reselecting the ROI with the “Oval” selection tool and the “Edit”, “Clear Outside” option (Fig. 2B). Subsequently, the color balance was adjusted for each image until only the vascular tracing was visible using the “Image”, “Adjust” and “Color Balance” option (Fig. 2C). Inaccuracies were covered using the “Paintbrush” with black color. Subsequently, the color channels were separated by opting for “Image”, “Color”, “Split channels” selection. The channel showing the vascular network in bright color with a dark background was selected. Afterwards the image was transformed to a binary color scheme (“Process”, ”Binary”, “Make binary”) (Fig. 2D) and skeletonised using the “Process”, “Binary” and “Skeletonize” option (Fig. 2E). The Skeleton was then analyzed with the Plugin “Analyze”, “Skeleton”, “Analyze Skeleton” function. After completion of analysis ImageJ provided a detailed image showing the vascular network (Fig. 2F) as well as a data table containing the “number of branches” (n), the “number of junctions” (n) as well as the individual “vessel length“ (PX) as relevant parameters.
3. The workflow associated time, required for tracing, image editing, and analysis was documented for each work step in every individual image and summed up to an individual time (in seconds) per image.

Identical images and ROI were also analyzed using the ROI analysis in the deep-learning based image analysis (DLBA) IKOSA CAM application (v3.2.0., Access Apr. 2024, cloud-based platform) without extra training^29,33^.

**Fig. 3:**
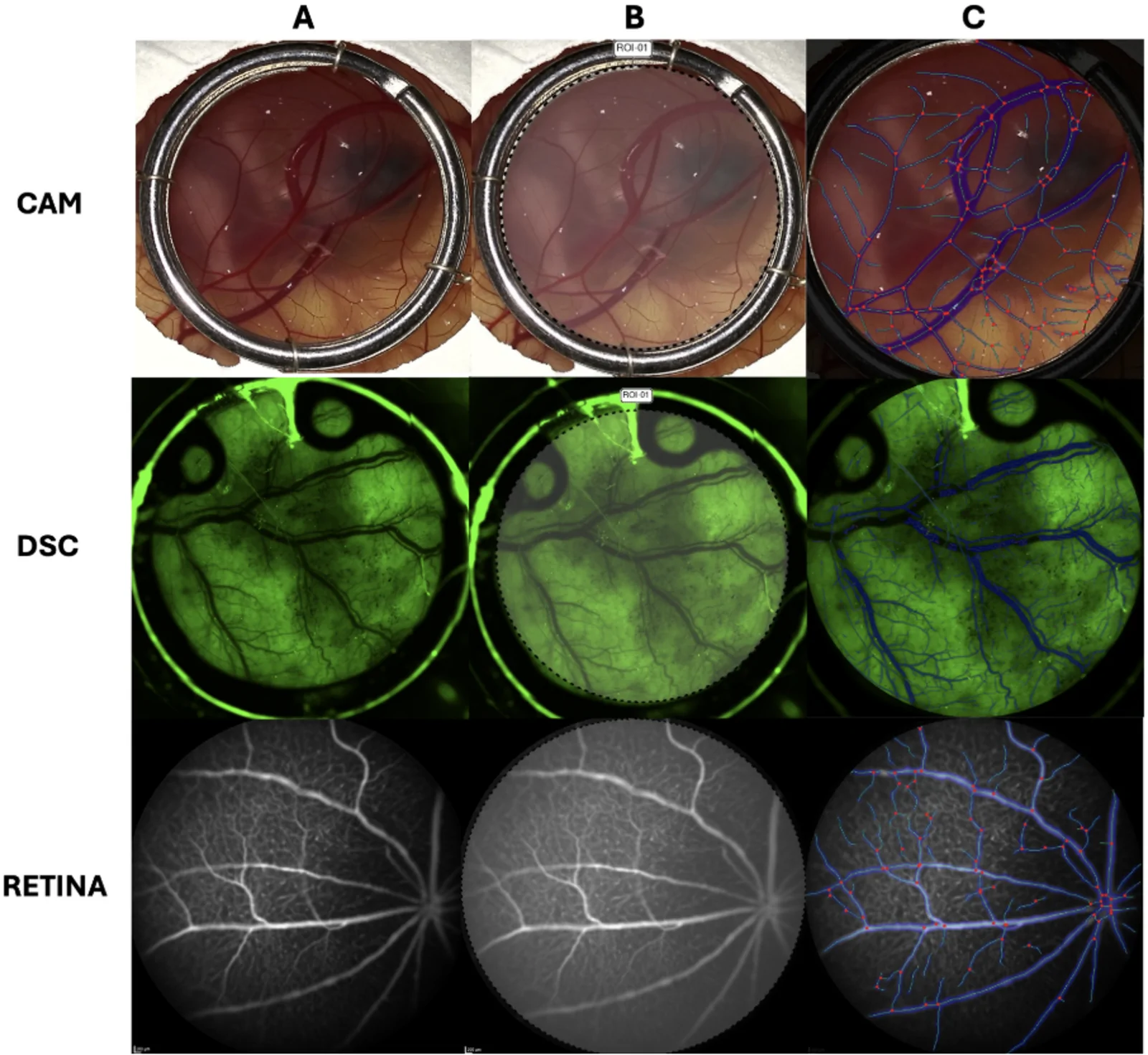
(**A**) Original image captured with microscope. (**B**) Region of interest (ROI) defined in using IKOSA. (**C**) Visualization of recognized vessels and branches by IKOSA.

Deep Learning-based vessel analysis (DLBA):

1. After creating a new project and uploading all relevant images each image was selected separately for further analysis (Fig. 3A). Subsequently, the “Annotations drawing mode” was selected and “Add circle region regions of interest” was chosen to define the ROI (Fig. 3B). After defining the ROI with the “Edit” function, “Submit image analysis” was selected. After completion of analysis IKOSA provided a detailed image showing the vascular network (Fig. 3C) as well as a data table containing the “total vessel area” (PX), “total vessel length”/vessel length (PX), “vessel mean thickness” (PX) and the “number of branching points” (n).
2. The workflow time required for the DLBA was documented for each work step in every individual image and summed up to an individual time (in seconds) per image. It is important to note, that the measured duration reflects the complete user workflow, including image upload, server communication, automated analysis, and result retrieval, rather than the isolated time needed for analysis by the DLBA. Different runs were performed with exactly the same images and settings.

Fig. 2 A shows an exemplary comparison of the marking by a rater, while Fig. 3C shows the counterpart provided by the DLBA.

### Statistical analysis of branching points and vessel lengths

After both manual analysis and DLBA, the number of junctions/branching points per ROI as well as the vessel length per ROI were transferred into Microsoft Excel files (Microsoft Corporation, Redmond, Washington, USA). Vessel length was factorized to align from pixels to millimeters. The above-mentioned values were then statistically compared with regard to differences between the three human raters and the DLBA. Statistical analysis was performed using Prism for Windows (version 10.6.0; GraphPad Software, California, USA).

Though of limited value for small groups, to compare findings from both manual analysis and DLBA for the number of junctions/branching points per ROI, vessel length per ROI and the time required for the analytical workflow was statistically analyzed. Due to the limited sample size, non-parametric statistical methods were selected a priori for all comparative analyses. Differences between groups were evaluated using the Friedman test due to the small sample sizes compared. Subsequently, a Bland-Altman method comparison was performed for each individual rater versus the DLBA. To further evaluate and characterize the image analysis results, a Wilcoxon matched-pairs signed-rank test was conducted between individual raters and the DLBA given. Again, due to the small sample size nonparametric tests were performed. As this study was designed as an exploratory validation study, no formal a priori sample size calculation was performed. The number of analyzed images was determined by the availability of representative datasets retrievable from the three established angiogenesis models, thus adhering to the principles of the Three Rs (Replacement, Reduction, and Refinement), thereby balancing scientific objectives with the ethical obligation to minimize the use of experimental animals^34^.

### Rating of image quality and vessel analysis

After completion of quantitative analysis images with the annotated vascular network were reevaluated. Subsequently, a blinded rating process was performed for every annotated image (DLBA & human annotation) by every rater. More specifically, each rater determined the accuracy of vascular annotation for every image. Rating was performed using a 6-Point Likert scale (1 = completely inaccurate; 2 = mostly aberrations; 3 = many aberrations; 4 = multiple aberrations; 5 = only slight aberrations; 6 = almost perfect). Moreover, aberrations were further specified by determining whether the specific entity marked too many (+ x) or too few (-x) vessels. Additionally, the image quality was rated using the same 6-Point Likert scale to determine any systematic bias due to bad source material. A statistical analysis of this rating data was again performed using a Wilcoxon matched-pairs test signed-rank test, due to the small number of samples and non-normally distributed datasets in the D’Agostino & Pearson test.

### Interrater reliability of vessel tracing by humans

Interrater reliability was assessed using the Intraclass Correlation Coefficient (ICC) for continuous vessel measurements (branching points and vessel length) from three fixed raters. Data was imported from Excel into Python (version 3.11.13) in the Google Colab environment and reshaped into long format for analysis with the pingouin package. A two-way mixed-effects model was applied to compute ICC(3,1) for single-rater reliability and ICC(3,k) for the reliability of the mean rating. Results are reported as ICC values with 95% confidence intervals, F-statistics, degrees of freedom, and p-values, using absolute agreement as the metric.

### Testing of alternative methodologies (AngioTool)

To evaluate an alternative method for vascular network analysis, we used the open-source software AngioTool to quantify the vascular networks for three images of each category (CAM, DSC, Retina). AngioTool is a Java-based software tool designed for quantitative analysis of vessel networks. Thus it allows quantitative assessment of various vessel morphometric and spatial parameters including vessel length and density, branching indices, among other parameters. The detailed workflow has previously been described^21^. Images were uploaded without additional editing and formatting and the threshold was adjusted to achieve the closest visual representation of the vascular network. Results were retrieved seconds later using the standard settings to allow maximum comparability.

## Results

### Vessel analysis for each entity

**Fig. 4:**
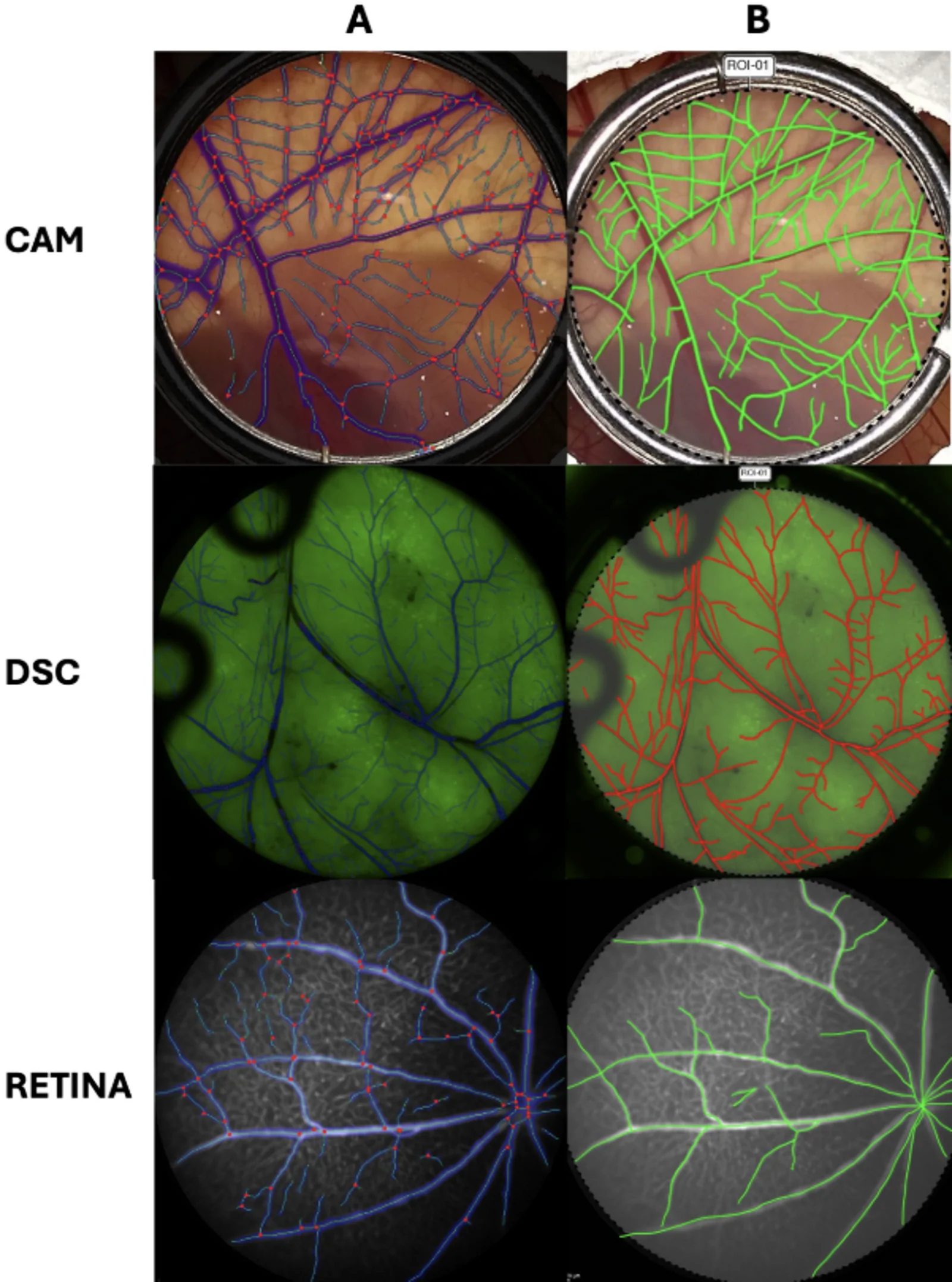
Exemplary tracing by DLBA and humans for each entity (CAM, DSC, Retina). (**A**) Vessel and junction tracing by DLBA. (**B**) Vessel and junction tracing by human rater.

**Fig. 5:**
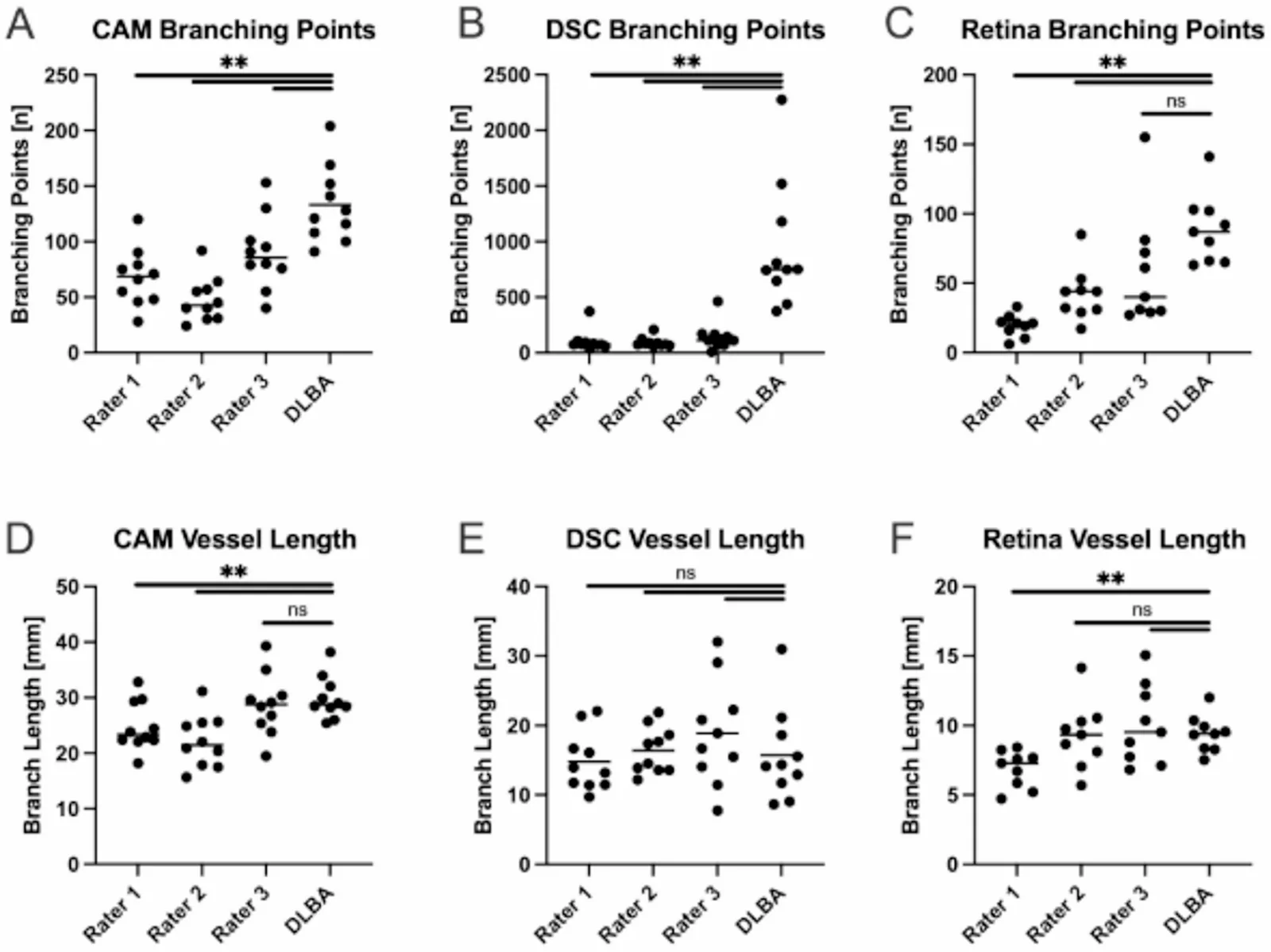
Results of vessel analysis for each entity are shown as strip plots, with individual values represented as points. The median for each group is indicated, and pairwise comparisons were performed using the Wilcoxon matched-pairs signed-rank test. Statistically significant differences are marked with asterisks (*).

Exemplary tracing as provided by DLBA and humans for each entity is visualized in Fig. 4. The results of the vessel analysis based onannotations provided by Raters 1–3 and the DLBA are shown in Fig. 5. While the raters and the DLBA determined similar values for vessel length, there are clear differences between raters and the DLBA in terms of branching points for DSC. Here, the result of Rater 1 for instance is ten times lower than the value provided by the DLBA (Table S1).

### Workflow time per analysis and ROI

**Fig. 6:**
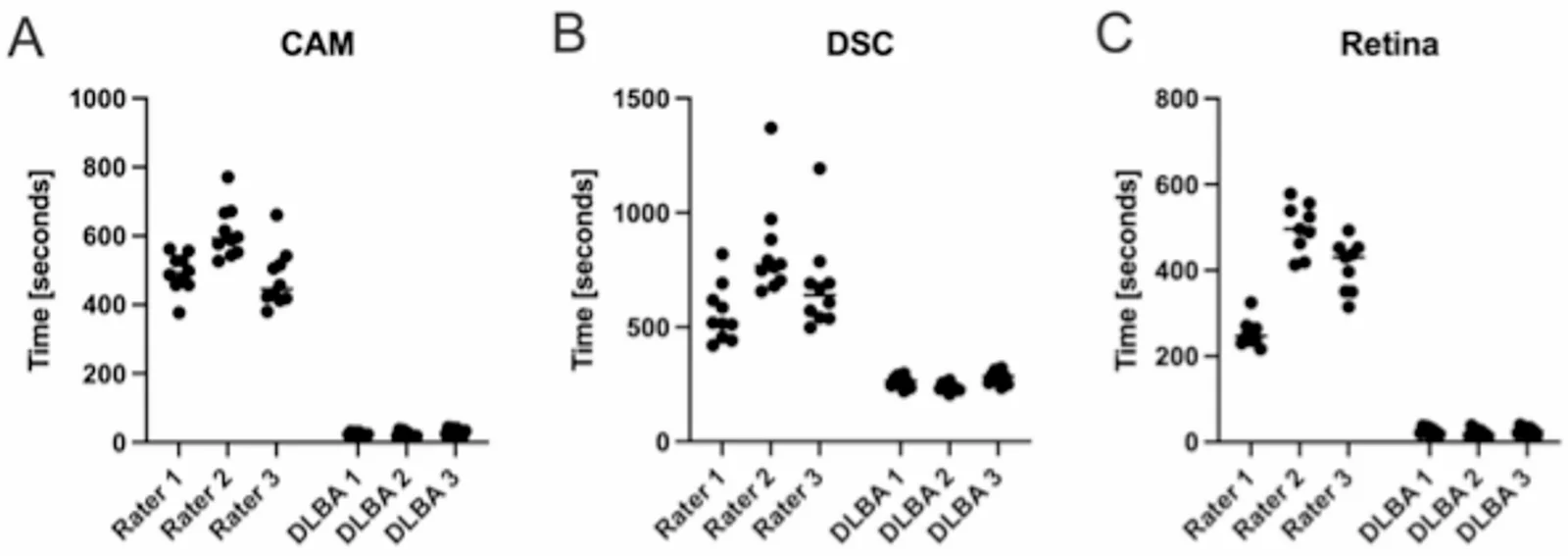
Workflow times for image tracing by the human raters (Raters 1–3) and for tracing and analysis by the DLBA are presented as scatter plots showing individual measurements.

The raters needed a mean of 526 s (95% CI 492, 561) to annotate the vessels of the CAM, 690 s (95% CI 611, 769) for the DSC, and 387 s (95% CI 341, 432) for the retina. The DLBA required a mean of 26 s (95% CI 23, 29) for the entire analysis (including vessel tracing, quantitative analysis, and result retrieval) for the CAM, 345 s (95% CI 282, 409) for the DSC, and 25 s (95% CI 21, 28) for the retina. Workflow times are shown in Fig. 6.

### Bland-Altman analysis

**Fig. 7:**
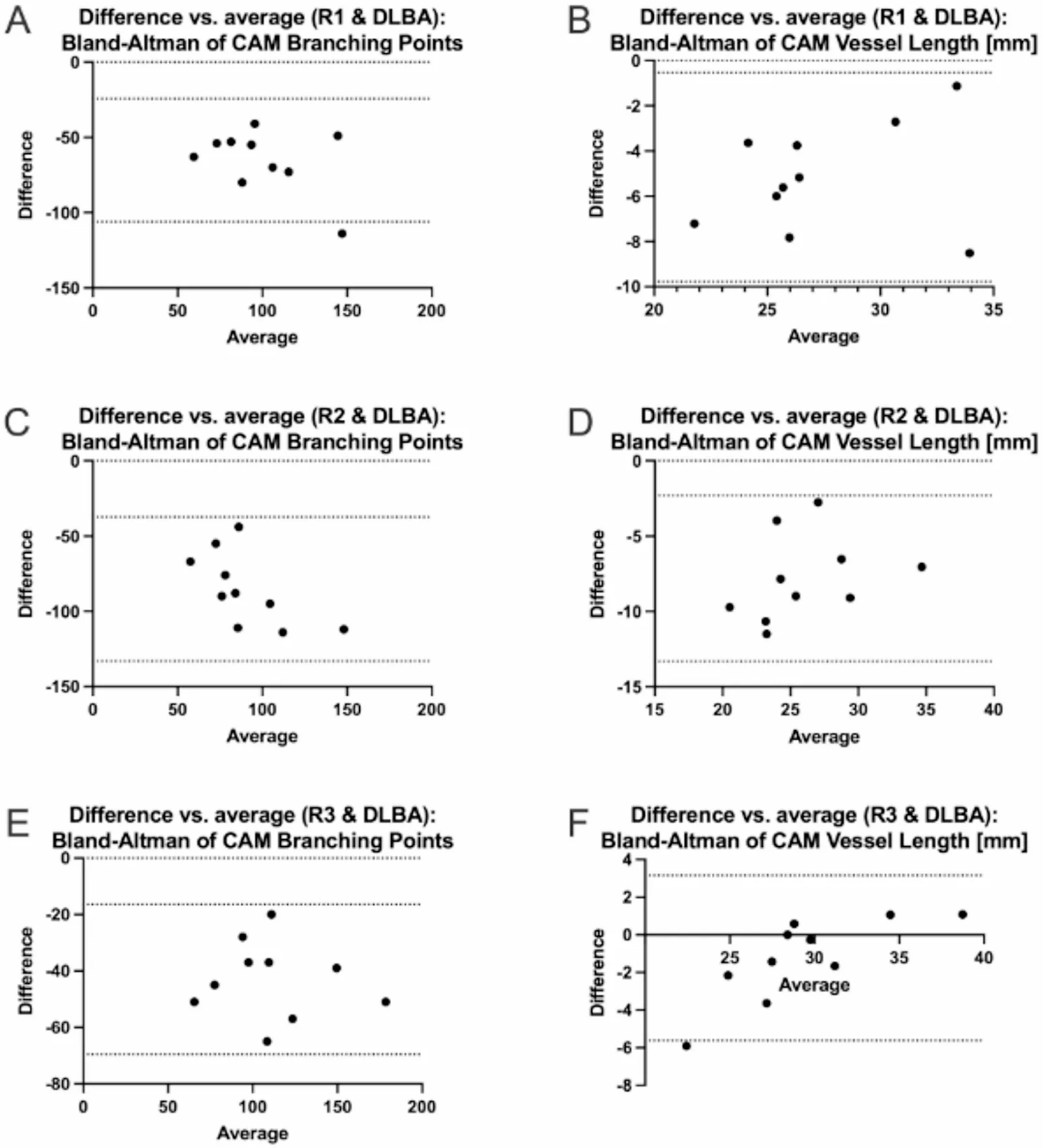
Bland-Altman plots comparing the agreement of manual annotation of three raters (R1–R3) with the DLBA results for CAM images. Differences and mean values for branching points (**A**,** C**,** E**) and vessel length (**B**,** D**,** F**) are shown. The DLBA systematically provides higher values than the raters for both parameters, with the deviations being more pronounced at the branching points.

Measurements of human raters and DLBA are compared using Bland-Altman plots for each entity. A Bland–Altman plot assesses the agreement between two measurement methods by visualizing the differences between their paired values against their mean. The small sample size of measurements should be considered when interpreting these plots. Fig. 7 shows Bland-Altman plots comparing the manual annotation of three raters (R1, R2, R3) with a deep learning-based analysis (DLBA) for the CAM. Differences between paired measurements are plotted against their mean values are shown for the number of branching points (Fig. 7A, C and E) and the mean vessel length (Fig. 7B, D and F). In all plots, the differences are predominantly in the negative range, indicating that DLBA systematically provides higher values than the raters for both branching points and vessel lengths. The deviations are more pronounced for branching points than for vessel lengths.

**Fig. 8:**
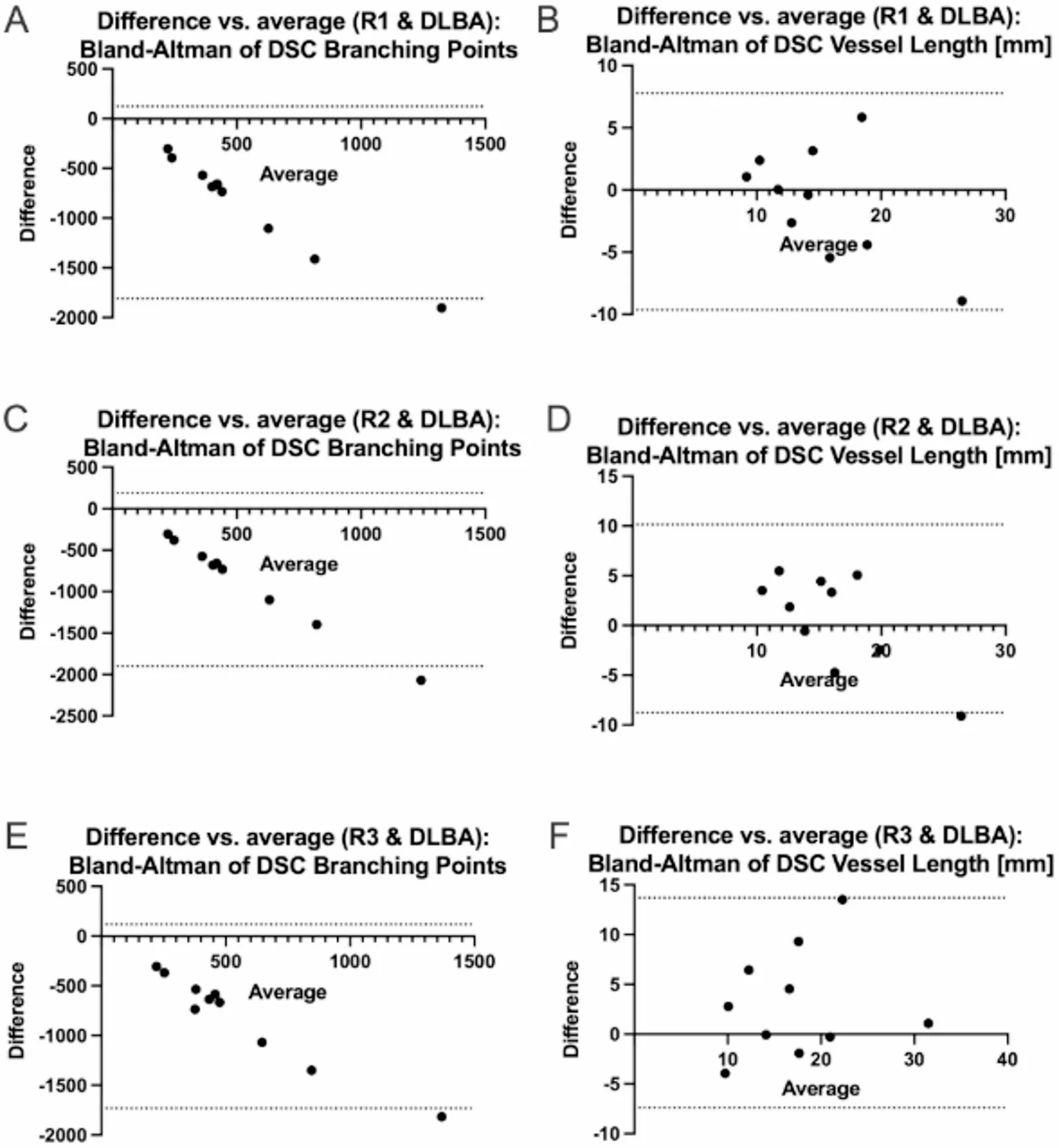
Bland-Altman plots comparing the agreement of manual evaluation of three raters (R1–R3) with the DLBA of the DSC. Differences and mean values for branching points (**A**,** C**,** E**) and vessel length (**B**,** D**,** F**) are shown. DLBA consistently detects more branching points than the raters, while smaller and inconsistent deviations are seen in vessel lengths. The downward trend for branching points indicates a systematic difference between the raters and the DLBA in the recording of branching points (**A, C, E**).

Figure 8 provides Bland-Altman plots comparing the DSC results of different entities. The differences from the mean values for the number of branch points (Fig. 8A, C, E) and the mean vessel length (Fig. 8B, D, F) are shown. For the branching points, the differences are clearly in the negative range, indicating that DLBA consistently detects a higher number of branching points than the raters. The downward trend for branching points indicates a systematic difference between the raters and the DLBA in the annotation of branching points. Regarding mean vessel length, on the other hand, there are both positive and negative differences, with the deviations being smaller overall and no clear systematic trend apparent. (Fig. 8B, D, F)

**Fig. 9:**
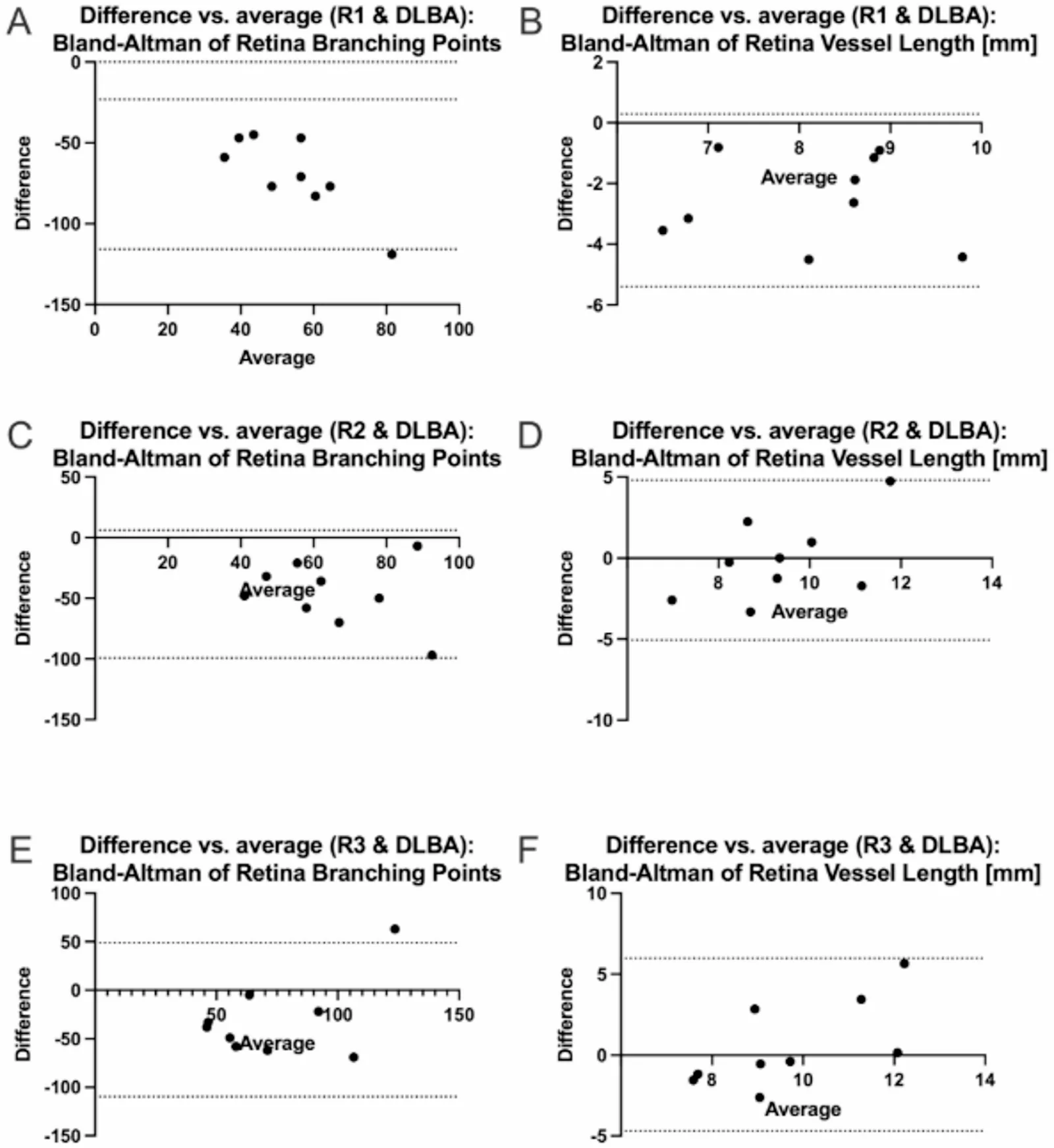
Bland-Altman plots comparing the agreement of manual evaluation of three raters (R1–R3) with DLBA of the retinal vasculature. Differences and mean values for branching points (**A**,** C**,** E**) and mean vessel length (**B**,** D**,** F**) are shown. DLBA consistently provides higher values for the number of branching points, while only minor and inconsistent deviations occur in vessel lengths.

Figure 9 visualizes the Bland-Altman plots for the retina. The differences from the mean values for the number of branching points (A, C, E) and the vessel length (B, D, F) are shown. In all plots for the branching points, the values are predominantly in the negative range, indicating that the DLBA consistently detects a higher number of branching points than the raters. For vessel lengths, there are smaller and sometimes variable deviations that do not show a clear systematic trend. Overall, the DLBA thus shows a robust quantification of the branching points with higher values on average.

### Rating of image quality and vessel tracing

To reevaluate the vessel tracing of the DLBA and humans, the annotated images were rated in a blinded manner using a six-point Likert scaleby three different raters. Image quality was also rated. Accordingly, Figure S1 visualizes the rating of image quality and vessel analysis for each entity (Rater 1, Rater 2, Rater 3, DLBA). Detailed median values together with interquartile range (IQR) are provided in Table S2. While both CAM and retina images received a median rating of 6 for image quality, the DSC received 5. Rater 1 obtained the highest median rating of 6 for each image entity (CAM, DSC, Retina), followed by the DLBA which received a median of 6 for CAM and Retina, while achieving a median of 5 for DSC. Rater 3 received the lowest rating with a median of 5 for every image entity. In their assessment, the raters could also indicate whether the entity had marked too few or too many vessels. While human vascular marking was mostly rated as too little or just right, the DLBA showed more heterogeneous patterns. For the CAM, the raters rated the DLBA annotation as accurate in almost all cases (neither too many nor too few vessels were marked). For DSC, the raters concluded that the DLBA marked too many vessels. Conversely, for the retinal images, the DLBA tended to mark too few vessels according to the raters.

### Interrater reliability of human vessel analysis

Interrater reliability for the human raters was assessed using a two-way mixed-effects model with absolute agreement. Table S3 shows both ICC3 (single-rater reliability) and ICC3k (reliability of the mean of all three raters) with 95% confidence intervals, F-statistics, degrees of freedom, and p-values.

For CAM and DSC measurements (branching points and vessel length), single-rater ICCs ranged from 0.66 to 0.78, indicating moderate to good reliability. Reliability was further improved when the mean of all three raters was considered, with ICC3k values between 0.86 and 0.92, reflecting good to excellent agreement.

For retina measurements, reliability varied between metrics. Vessel length showed good single-rater reliability (ICC3 = 0.74) and excellent mean-rater reliability (ICC3k = 0.90). Branching points, however, exhibited lower single-rater reliability (ICC3 = 0.43), and moderate reliability for the mean of three raters (ICC3k = 0.70), indicating greater variability among raters for this metric.

All ICCs were statistically significant (*p* < 0.05), confirming that the measured features were reliably assessed by the human raters.

### Testing of alternative methodologies (AngioTool)

Following the reviewer’s suggestion, AngioTool was additionally evaluated as an open-source baseline for vessel segmentation. While only taking around 35 s per image, visual assessment revealed insufficient segmentation quality in the CAM and DSC datasets, precluding a meaningful quantitative comparison with manual annotation and the DLBA approach. Although segmentation performance was comparatively better for the retinal images than CAM and DSC images, the annotation quality failed to produce segmentations of sufficient quality for meaningful quantitative analysis compared to the reference annotations of both manual annotation and DLBA. Representative segmentation results are provided in the supplementary Figure S3. Owing to the insufficient segmentation performance, no further quantitative analyses using AngioTool were performed.

## Discussion

Even though many studies report on the effects of molecules and therapies on vascular networks^3,12–14,18^, and consensus recommendations are available, a well standardized methodology for quantification of vascular networks is still missing^35^. Different vessel analysis software ranging from Python based scripts specialized for mouse retina^36^ to the universal AngioTool^21^ or the IKOSA CAM DLBA exist. However, little research has been conducted on evaluating the workflow of DLBA against the current gold standard of human tracing^24,27,37^. In this study, image analysis proved to be successful with both manual tracing and DLBA respectively for all three angiogenesis models. However, for all entities analysis showed individual strengths and limitations.

### Human vessel tracing

Although manual tracing was performed using the same methodology, training and annotation protocols for all three raters, significant differences among most rater pairs (R1 vs. R2; R1 vs. R3; R2 vs. R3) were present, thus indicating a high variability in manual tracing (Fig. 6). Reconsideration of the annotations showed that the threshold for consideration as a countable vessel varied between all three raters but remained relatively consistent throughout the analysis, indicating a consistent, rater-specific methodological bias between the three raters. The ratings given for human tracing ranged from 5 to 6 for each image entity (CAM, DSC, Retina) revealing an overall sactisfactory rating of tracing quality (Fig. S1). Human vessel tracing was mostly rated as either too low or just right among raters. This “limitation” could possibly be overcome by a more rigorous definition of countable vessel sizes before annotation. On the other hand, implementing these clear definitions for vessel tracing is challenging. For instance, measuring vessel diameters to ensure sufficient marking is feasible, yet it is also a very time-consuming process when carried out for large vascular networks. It should be noted that manual vessel annotation, while commonly used as the reference standard in angiogenesis research, is thus inherently subject to inter-observer variability and lacks universally accepted annotation criteria for vessel inclusion and branching point definition. Similar challenges have been reported for medical image analysis in scientific literature. However, expert-derived annotations are often used as reference standards despite known observer-dependent variability^26^. Consequently, the present study should be interpreted as an assessment of agreement between manual and AI-assisted quantification methods rather than a determination of absolute accuracy.

The interrater reliability of the human raters was also assessed (Table S3). For the CAM and DSC measurements (branching points and vessel length), the single-rater intraclass correlation coefficients (ICC3)^38^ ranged from 0.66 to 0.78, indicating moderate to good reliability. However, while vessel length for retina measurements showed good single-rater reliability (ICC3 = 0.74), branching points exhibited a lower single-rater reliability (ICC3 = 0.43). This underscores the postulated limited reproducibility of human tracing also described for similar models^39^. Obviously, identification of vasculature with the described iPad-based annotation method has limitations and could be refined by using high magnification as well as pixel-level tools to annotate images with pixel-wide centerlines. However, more refined annotation methods at high magnification also make the methodology prone to mis-segmentation due to loss of oversight. Furthermore, pixel-level annotation of highly magnified segments of the vascular network will likely be traded for a substantial increase in annotation time. Nevertheless, future studies using fully accessible segmentation outputs could additionally incorporate pixel-wise metrics.

### DLBA in vessel analysis

In contrast, DLBA showed perfect reproducibility, yielding identical results up to the last decimal digit if the identical ROI was selected. The tested IKOSA DLBA runs a fixed, pre-trained segmentation model inside a controlled inference pipeline (fixed preprocessing, model weights, and postprocessing) resulting in bit-for-bit identical outputs for repeated analyses of the same image^40^. Within this study, the DLBA performed most accurately on the CAM assay images which seems reasonable since it was trained on both retinal fundus images and images of the CAM ^27,31,32^(Figs. 2 and 3). Overall, the DLBA seems to overestimate both branching points and vessel length when compared to human tracing. Especially in the DSC, and as shown in detail in Figure S4 , areas of over-segmentation often occur in areas where very thick vessels are present. Whilst manual annotation recognizes the vessel as one, the DLBA overinterprets the structure by counting a complex network when vessels are very large. Also, the discrepancy in branching points might be explained by the occasional double recognition of branching points by the DLBA (Fig. 4A & Fig. S4). On the other hand, the highest agreement between DLBA and human tracing was shown for vessel length (Figs. 5 and 7). Beyond the default use case, IKOSA performed worse for DSC and retina images (Figs. 3 and 4). Especially the branching points identified by the DLBA differed substantially from those marked by the raters when it came to the DSC (Fig. 5B). The difference between DLBA and Rater 1 was as high as a factor of ten (Table S1) and can likely be attributed to the above-mentioned misannotation over larger vessels (Fig. S4). However, the values for vessel length were of a similar magnitude between human tracing and DLBA. Bland-Altman plots for DSC branching points visualized a downward trend for branching points indicating a systematic difference between the raters and the DLBA in the annotation of branching points (Fig. 8A, C, E). A closer evaluation of the DLBA results revealed mislabeling of artifacts as vessels and showed multiple recognition of branching points (Fig. S4). The same pattern was observed for the retinal images, although the deviations were not nearly as pronounced. Here, a strong divergence in the branching points could be observed, with a high degree of consistency in the vessel length (Fig. 5C, F). Overall, except for DSC vessel length, the DLBA yielded the highest numbers for each entity and category (Table S1). Nonetheless, the overall quality of vessel recognition was still very good, represented by a median rating of 5 [IQR 4.75–6] for DSC and a median rating of 6 [IQR 5–6] for the marking of vessels provided by the DLBA (Table S2).

### Comparison of tracing methods

Given the high reproducibility, a systematic tracing bias may be less problematic for relative comparisons within a consistent experimental workflow. Accordingly, potential pro- and anti-angiogenic effects may still be detectable in the experimental setting. In contrast, since a pairwise comparison between the three evaluators’ annotations (R1 vs. R2; R1 vs. R3; R2 vs. R3) showed significant differences for most combinations and each image type (CAM, DSC and Retina [Fig. 5]), one could argue that the analysis of an entire experiment should ideally be performed by the same rater in order to successfully detect effects between experimental groups.

Previous studies comparing manual evaluation with automatic evaluation do not report on interrater reliability. However, Kumawat et al. stated moderately strong correlation between the automated and manual macular vessel density in diabetic patients. The authors tested two different automatic image segmentation methods. While the reported *Otsu method* (a thresholding technique for optimal image segmentation based on maximizing inter-class variance) showed significantly lower values (*P* < 0.001) for manual analysis, the described *Huang method* (an adaptive thresholding technique that utilizes the image histogram for optimal threshold determination by minimizing intra-class variance) showed similar values regarding manual and automated measures^24^.

Regarding mice retina, Chen et al. report a mean *Dice coefficient* for all manually segmented and automated vessel maps of 0.75 ± 0.27 for a held-out test set and 0.77 ± 0.17 for an external test set. The *Dice coefficient* represents a statistical measure used to evaluate the similarity between two sets, commonly applied in image segmentation to assess the overlap between predicted and ground truth labels. The authors discussed possible reasons for the mismatch. They referred to the identification of vessels by the AI algorithm that were not marked by the grader, and vice versa, along with differences in tortuosity between manually and automatically segmented vessels^37^. Faihs et al. evaluated IKOSA and benchmarked its performance against AngioTool and manual tracing for the analysis of CAM vessels. Here, for branching points, the authors report the closest agreement with manual counts (mean difference 12.18) for IKOSA. In contrast, AngioTool significantly overestimated branching points (mean 944.26 vs. 190.61 manually). According to the authors, differences between methods increased with higher branching density. Visual inspection of segmentation masks confirmed the reliability of the IKOSA analysis. For total vessel length, the authors reported that AngioTool consistently reported higher values than IKOSA, particularly in images with extensive vascular networks (mean difference ~ 30,049 Px). These findings highlight systematic differences between analysis methods for both branching points and vessel length, with IKOSA providing results closer to manual assessment^27^. In our experience, AngioTool, at least for the images analyzed by our working group, was substantially inferior in terms of segmentation quality when compared to both manual annotation and DLBA (Fig. S3). Editing of the inserted images by enhancing the contrast may have produced better results but is also associated with additional workflow time and the potential to reduce reproducibility and comparability of analysis. The exploratory evaluation of AngioTool should be regarded as a feasibility assessment rather than a formal performance comparison. Under the default workflow, AngioTool did not generate vessel segmentations of sufficient quality for reliable quantitative analysis of the investigated datasets. According to the software developer, the IKOSA CAM application performs only application-specific preprocessing required for technical compatibility with the AI model (e.g., bit-depth conversion), whereas meaningful application of AngioTool would likely have required additional user-dependent image optimization, reducing the methodological comparability between the workflows.

Literature research confirms significant discrepancies between DLBA and human annotation. Certainly, using an algorithm specially trained for the corresponding experiment is the ideal solution. If not available, using pre-existing algorithms may be justifiable. However, researchers should double-check or even benchmark the analysis on representative samples with their own manual analysis before building the entire experimental analysis on the results. Slight inaccuracies should be carefully noted but may be justified considering the high reproducibility of the results, especially since human evaluation is not flawless either.

### Time efficiency of vessel analysis (Workflow time)

Time efficiency can be considered the most relevant aspect favoring the DLBA over manual tracing, especially in experimental setups comparing large groups. The time burden increases rapidly, especially when all images are otherwise analyzed by a single person in order to avoid inter-rater variability as stated above. Across all three models, the DLBA consistently required substantially less workflow time per image than manual tracing (see Results and Fig. 6). Cumulatively, this amounted to approximately 195 min (≈ 11,700 s) saved per rater across the 29 analyz ed images.

### Implications of the study

The small number of images analyzed and the involvement of only three raters are obvious limitations of the present study. Hence, while the low number of raters reflects the reality in most laboratories and published research, it remains a methodological flaw. Yet, as our study was conducted as a validation study evaluating the workflow it may be justifiable in this case. Furthermore, as differences were apparent even with this small number of raters, the study highlights the importance of having all images evaluated by the same rater to detect small differences between experimental groups. While the DLBA provides various parameters including “total vessel area” (PX) and “vessel mean thickness” (PX), in this study only branching points (n) and vessel length (mm) were compared to human analysis. However, this is mainly due to the fact that the vessel thickness is very complicated to measure manually. Human annotation was performed according to the laboratory standard without a rigorous consensus definition of the vessel size considered eligible for annotation. Obviously, image quality can influence the quality of the analysis. In the present study, image quality was very good, as confirmed by the rating in Figure S1. Furthermore, the image quality showed only very low to moderate correlation with the subsequent rated entities (Fig. S2).

Although one human rater received the highest overall ratings, outperforming both the DLBA and the other raters, the use of the DLBA offers numerous advantages. Firstly, in addition to branching points and vessel length, DLBA offers further quantitative measurements (“total vessel area” (PX) and “vessel mean thickness” (PX)) that can provide value for analysis of pro- or antiangiogenic effects. Secondly, DLBA offers high reproducibility, whereas the raters showed consistant, and in most cases, significant differences in pairwise comparisons among raters (R1 vs. R2; R1 vs. R3; R2 vs. R3). This becomes a particular problem when different raters are involved in a project. The consensus paper for the use and interpretation of angiogenesis assays acknowledges the relevance of DLBA use^35^. Yet they also recommend double-checking or even benchmarking which would annihilate the key advantage of time efficiency but could be performed for random samples. From our perspective, time efficiency is the obvious advantage of the DLBA tracing providing a much higher quantitative output while maintaining a high level of consistency and reproducibility. Due to the high reproducibility of the errors, the use of DLBA for DSC and the retina may be justifiable. Additionally, the high reproducibility facilitates an option for repetitive analysis of the same object (CAM, DSC, or retina) at different time points.

It however has to be stated, that while deterministic inference ensures excellent reproducibility within a given software version, it does not preclude the presence of systematic algorithmic bias. Thus, the proprietary nature of closed-source software limits transparency regarding algorithmic modifications, and future software updates may affect quantitative outputs without being readily apparent to the end user. Therefore, periodic validation against established reference methods remains essential when implementing AI-assisted image-analysis workflows. Obviously, inclusion of additional open-source vessel-analysis tools could have provided further comparative information when evaluating the workflow of the DLBA. Yet, AngioTool, which was identified as a commonly used vessel-analysis tool in our literature search , proved to be inappropriate for the data analyzed within our study. Furthermore, the working group of Faihs et al. already evaluated IKOSA against other established CAM image-analysis approaches and reported robust and reproducible vessel quantification performance, highlighting its suitability for automated vascular analysis^27^.

The hybrid manual pipeline (iPad tracing – IrfanView color reduction – ImageJ skeletonization – Analyze Skeleton) introduces potential distortions at each step. Additionally, the color-processing steps applied specifically to retinal images create an asymmetry relative to the DLBA workflow. This may indeed contribute to the comparatively poor ICC for retinal branching points and represents a valid alternative explanation beyond inter-rater disagreement alone. Moreover, manual tracing may be improved by using pixel-level tools and at high magnification as it allows better manually defined vessels. Yet the specific images used are often auto-merged multi-image-arrays spanning thousands of pixels across. Annotating each vessel at high magnification pixel by pixel may therefore be feasible, yet it makes the methodology prone to missegmentation and would expand the required time to an almost impractical degree. As an exploratory pilot validation study, the present work was not designed or statistically powered to evaluate the influence of image characteristics such as vessel density, contrast, or image quality on automated quantification. Future studies including edited images under varying imaging conditions, larger and more heterogeneous datasets will be required to address these aspects. The present analysis is limited to morphometric parameters and does not capture molecular aspects of angiogenesis. Future validation approaches could therefore combine image-based vessel quantification with histological analysis^41^ or molecular readouts of angiogenesis-related markers, such as VEGF gene expression analysis^41^ or VEGF protein quantification by ELISA^42^.

## Conclusion

Vascular analysis across different experimental models is highly relevant in basic research. Yet, a standardized methodology has not yet been established. This study revealed that the tested DLBA IKOSA CAM provides a time-efficient and reproducible tool with good agreement with manual tracing of vascular networks on the CAM. Despite some limitations of the assessed IKOSA CAM DLBA in the analysis of vascular networks of the retina and DSC, the software provided a standardized high-quality analysis of vascular networks. Its time efficiency allows a high quantitative output, facilitating repetitive longitudinal analysis. Furthermore, the evaluated DLBA, IKOSA CAM, provided a simple and intuitive application without requiring any coding skills. In conclusion, this pilot validation study supports the use of AI-assisted vessel analysis as an efficient and reproducible approach for vascular quantification. While systematic differences between manual and automated analyses should be considered, particularly for branching point quantification, the substantial reduction in analysis time together with the overall agreement observed in this study highlights the potential of AI-assisted workflows to complement conventional manual vessel analysis. Further validation in larger datasets and additional angiogenesis models will help to define the optimal scope of application of such tools. Due to the high relevance regarding standardization of research findings, intuitive open-source solutions are desirable. In scientific research, if non-standardized workflows are used, systematic comparability across independent studies, laboratories, different image analysis platforms, or successive versions of commercial software may deeply affect the comparability and reproducibility of quantitative measurements. Thus, the establishment of comparable standards is of utmost importance.

## Supplementary Information

Below is the link to the electronic supplementary material.


Supplementary Material 1


## Data Availability

The datasets generated and analyzed during the current study are available from the corresponding author upon reasonable request.
