# Supplementary material for: Workflow evaluation of a commercial deep learning-based image analysis tool for the quantification of angiogenesis in vivo
Source: Sci Rep. 2026 Aug 28;16:27110. doi: 10.1038/s41598-026-68133-1 (PMC13524943; doi:10.1038/s41598-026-68133-1)
Supplement: Supplementary file 1 [file 41598_2026_68133_MOESM1_ESM.docx]

**Supplementary Material**

Table S1: Quantitative results of vessel analysis provided by raters and DLBA

|  | **Rater 1** | **Rater 2** | **Rater 3** | **DLBA** |
| --- | --- | --- | --- | --- |
| **Branching points [n]** | | | | |
| **CAM** | **68.50** [47.50-81.75] | **42.50** [30.75-58.75] | **85.50** [70.75-108.3] | **124.5** [106.0-156.3] |
| **DSC** | **75.50** [67.00-95.75] | **79.00** [68.50-97.5] | **114.5** [69.00-166.3] | **750.0** [594.3-1265] |
| **Retina** | **21.00** [13.00-24.00] | **44.00** [30.00-49.00] | **40.00** [29.50-76.50] | **87.00** [65.50-102.5] |
| **Vessel length [mm]** | | | | |
| **CAM** | **23.35** [22.27-29.40] | **21.46** [17.76-25.52] | **28.75** [24.97-31.52] | **28.75** [27.64-32.50] |
| **DSC** | **13.56** [11.47-17.86] | **15.96** [13.58-19.15] | **17.78** [13.41-23.94] | **14.24** [11.04-19.24] |
| **Retina** | **7.28** [5.53-7.96] | **9.34** [7.57-10.41] | **9.52**  [7.43-12.57] | **9.39** [8.32-10.14] |

Results shown as Median [IQR]

Table S2: Rating of image quality and vessel annotation

|  | **Image quality** | **Rater 1** | **Rater 2** | **Rater 3** | **DLBA** |
| --- | --- | --- | --- | --- | --- |
| **CAM** | **6** [5.75 - 6] | **6** [5 - 6] | **5** [4 - 5] | **5** [4 - 5] | **6** [6 - 6] |
| **DSC** | **6** [5 - 6] | **6** [5 - 6] | **6** [5 - 6] | **5** [5 - 6] | **5** [4.75 - 6] |
| **Retina** | **5** [5 - 6] | **6** [5 - 6] | **5** [5 - 6] | **5** [5 - 5] | **6** [5 - 6] |

Results shown as Median [IQR]

Table S3: Interrater-reliability of human vessel analysis (Two-Way Mixed Effects, absolute agreement)

|  | Type | ICC | CI 95% | pval |
| --- | --- | --- | --- | --- |
| CAM  branching points | ICC3 | 0.664625 | [0.31, 0.89] | 0.000258 |
|  | ICC3k | 0.856016 | [0.58, 0.96] | 0.000258 |
| CAM  vessel length | ICC3 | 0.779498 | [0.5, 0.93] | 0.000008 |
|  | ICC3k | 0.913832 | [0.75, 0.98] | 0.000008 |
| DSC  branching points | ICC3 | 0.783970 | [0.5, 0.93] | 0.000007 |
|  | ICC3k | 0.915874 | [0.75, 0.98] | 0.000007 |
| DSC  vessel length | ICC3 | 0.784938 | [0.51, 0.94] | 0.000006 |
|  | ICC3k | 0.916314 | [0.75, 0.98] | 0.000006 |
| Retina  branching points | ICC3 | 0.433199 | [0.02, 0.81] | 0.020286 |
|  | ICC3k | 0.696313 | [0.05, 0.93] | 0.020286 |
| Retina  vessel length | ICC3 | 0.743292 | [0.41, 0.93] | 0.000074 |
|  | ICC3k | 0.896763 | [0.68, 0.97] | 0.000074 |
| **Interpretation guidelines (Koo & Li, 2016 ^34^)**  < 0.50 → poor  0.50 – 0.75 → moderate  0.75 – 0.90 → good  > 0.90 → excellent | | | | |

ICC = Intraclass Correlation Coefficient; ICC3 = single-rater, two-way mixed-effects (absolute agreement); CC3k = corresponding average-measures ICCs; CI95% = 95% confidence interval; pval = p-value.

Figure S1: Results of image-quality ratings and entity-specific ratings are shown as box plots, with whiskers indicating the minimum and maximum values. Pairwise comparisons were performed using the Wilcoxon matched-pairs signed-rank test, as non-normal distribution was assumed based on the D'Agostino & Pearson test. Significant differences are indicated by asterisks; non-significant comparisons are labeled “ns”.

Figure S2: Results of Rating for Image quality and each entity. Individual values shown as box plots. Pairwise comparisons were performed using the Wilcoxon matched-pairs test signed rank test as non-Gaussian distribution was assumed (D'Agostino & Pearson test). Corresponding Spearman correlation of all rated entities illustrated with heatmaps.

Figure S3: Exemplary images of AngioTool annotated images of Retina (1), DSC (2) and CAM (3)

Figure S4: Exemplary high-magnification image of the DSC highlighting the mis-segmentation of both methods. 1: DLBA, 2:Manual annotation. A: Original image, B: Annotated image
